# Welcome to Epidemiology and Health

**DOI:** 10.4178/epih/e2009001

**Published:** 2009-10-29

**Authors:** Bo Youl Choi

## Abstract

The Korean Society of Epidemiology publishes a scholarly journal titled 'Korean Journal of Epidemiology', which announces and discusses the results of epidemiological studies from the past 30 yr. Since its first publication in 1979, the journal has contributed to the advancement of epidemiology as well as the prevention and control of disease, and the promotion of health in Korea.

In 2009, the editorial board has decided to publish the journal in English to contribute internationally, and change the journal's name. The new name of the journal is 'Epidemiology and Health'.

The abstract and full text of articles will be published as an open access online journal, which will be posted onto the homepage (http://www.e-epih.org/) in real time for anyone in the world to access free of charge. Our editorial policy is that 'Epidemiology and Health' is open to every researcher in fields related to epidemiology, regardless of membership, his or her major and nationality.

Editorials, lectures, review papers, original articles, epidemic and case investigations, brief communications and letters will be published to generate active discussion through the journal along with the publication of the papers.

'Epidemiology and Health' welcomes articles from various fields of epidemiology, such as 1) infectious diseases epidemiology, 2) chronic diseases epidemiology, 3) nutritional epidemiology, 4) clinical epidemiology, 5) pharmacoepidemiology, 6) genetic or molecular epidemiology, 7) social epidemiology, 8) environmental or occupational epidemiology, 9) epidemiological methods and biostatistics, 10) disease prevention and control, 11) health promotion and, 12) all other fields related to epidemiology.

